# A neuro-inspired general framework for the evolution of stochastic dynamical systems: Cellular automata, random Boolean networks and echo state networks towards criticality

**DOI:** 10.1007/s11571-020-09600-x

**Published:** 2020-06-11

**Authors:** Sidney Pontes-Filho, Pedro Lind, Anis Yazidi, Jianhua Zhang, Hugo Hammer, Gustavo B. M. Mello, Ioanna Sandvig, Gunnar Tufte, Stefano Nichele

**Affiliations:** 1 Department of Computer Science, Oslo Metropolitan University, Oslo, Norway; 2 Department of Computer Science, Norwegian University of Science and Technology, Trondheim, Norway; 3 Department of Neuromedicine and Movement Science, Norwegian University of Science and Technology, Trondheim, Norway; 4 Department of Holistic Systems, Simula Metropolitan, Oslo, Norway

**Keywords:** Dynamical systems, Implementation, Reservoir computing, Evolution, Criticality

## Abstract

Although deep learning has recently increased in popularity, it suffers from various problems including high computational complexity, energy greedy computation, and lack of scalability, to mention a few. In this paper, we investigate an alternative brain-inspired method for data analysis that circumvents the deep learning drawbacks by taking the actual dynamical behavior of biological neural networks into account. For this purpose, we develop a general framework for dynamical systems that can evolve and model a variety of substrates that possess computational capacity. Therefore, dynamical systems can be exploited in the reservoir computing paradigm, i.e., an untrained recurrent nonlinear network with a trained linear readout layer. Moreover, our general framework, called EvoDynamic, is based on an optimized deep neural network library. Hence, generalization and performance can be balanced. The EvoDynamic framework contains three kinds of dynamical systems already implemented, namely cellular automata, random Boolean networks, and echo state networks. The evolution of such systems towards a dynamical behavior, called criticality, is investigated because systems with such behavior may be better suited to do useful computation. The implemented dynamical systems are stochastic and their evolution with genetic algorithm mutates their update rules or network initialization. The obtained results are promising and demonstrate that criticality is achieved. In addition to the presented results, our framework can also be utilized to evolve the dynamical systems connectivity, update and learning rules to improve the quality of the reservoir used for solving computational tasks and physical substrate modeling.

## Introduction

Every day, humans produce exabytes of data and this trend is growing due to emerging technologies, such as 5G and the Internet of Things (McAfee et al. 2012). Given that the main computing technology is based on von Neumann architecture, the analysis of enormous amounts of data is challenging even for the popular deep learning methods (Oussous et al. 2018). Deep learning is a powerful data analysis tool, but it has some problems, including high energy consumption, and lack of scalability and flexibility. Therefore, a new type of architecture may be required to alleviate such problems, in particular energy efficiency, scalability, adaptability, and robustness. The brain, or rather, an architecture inspired by the brain, can be this new architecture. This computing organ is energy efficient, adaptable, robust, and can perform parallel processing through local interactions (Markram et al. 2011).

Artificial systems with similar dynamical properties to the brain exist, such as cellular automata (Wolfram 2002), random Boolean networks (Gershenson 2004), and artificial neural networks (Jaeger and Haas 2004; Maass and Markram 2004). However, their dynamics are difficult to program or control in order to perform useful computation. In such systems, Langton (1990) suggests that computational properties are connected to the “edge of chaos” behavior, a range of dynamical behaviors between order and disorder. In other words, they are systems critically near a phase transition. If the attractors of the system are in the critical state, this characteristic is called self-organized criticality (Bak et al. 1987). Systems with self-organized criticality have a common feature, i.e., power-law correlations in time or space that extend over several scales. Moreover, biological neural networks have been shown to self-organize into criticality, which is evaluated by the power-law distribution of neuronal avalanches (Heiney et al. 2019; Tetzlaff et al. 2010; Yada et al. 2017). Another important aspect of the computation performed in a dynamical system is the trajectory of system states traversed during the computation (Nichele and Tufte 2010). Such a trajectory may be guided by system parameters (Nichele and Tufte 2012).

**Table 1:** Examples of dynamical systems

Dynamical system	State	Time	Connectivity
Cellular automaton	Discrete	Discrete	Regular
Coupled map lattice	Continuous	Discrete	Regular
Random Boolean network	Discrete	Discrete	Random
Echo state network	Continuous	Discrete	Random
Spiking cellular automaton	Discrete	Continuous	Regular
Lattice differential equations	Continuous	Continuous	Regular
Liquid state machine	Discrete	Continuous	Random
ODEs in complex topology	Continuous	Continuous	Random

Table 1 presents some computing systems that are capable of giving rise to the emergence of complex dynamics. The approaches in such a table (and the work presented herein) are extensions to previous works (Pontes-Filho et al. 2019a, b). Dynamical systems with complex behavior can be availed by reservoir computing, which is a paradigm that resorts to dynamical systems to simplify complex nonlinear data. Such simplification means that reservoir computing utilizes the nonlinear dynamical system to perform a nonlinear transformation from nonlinear data to higher dimensional linear data. Such linearized data can be applied in linear machine learning methods which are faster for training and computing because they have less trainable variables and operations. Hence, reservoir computing is more energy efficient than deep learning methods and it can even yield competitive results, especially for temporal data (Schrauwen et al. 2007; Tanaka et al. 2019). Basically, reservoir computing exploits a dynamical system that possesses the echo state property and fading memory, where the internals of the reservoir are untrained and the training only happens at the linear readout stage (Konkoli et al. 2018).

Reservoir computers are most useful when their substrates’ dynamics are at the “edge of chaos” (Langton 1990). A simple computing system used as a reservoir is a cellular automaton (CA) (Nichele and Gundersen 2017; Nichele and Molund 2017). A CA consists of a grid of cells with a finite number of states that change according to simple rules depending on the neighborhood and own state in discrete time-steps. Other systems can also exhibit similar dynamics. The coupled map lattice (Kaneko 1992) is very similar to CA, the only exception is that the coupled map lattice has continuous states which are updated by a recurrence equation involving the neighborhood. A random Boolean network (RBN) (Gershenson 2004) is a generalization of CA where random connectivity exists. An echo state network (ESN) (Jaeger and Haas 2004) is an artificial neural network (ANN) with random topology. A spiking cellular automaton (Bailey 2010) is a CA whose cells are spiking neurons that communicate through discrete-events (spikes) over continuous time. A spiking neuron is a model of the biological neuron found in the brain. A lattice of ordinary differential equations (Chow et al. 1996; Larter et al. 1999) is a cellular automaton where state and time are continuous and updated by ordinary differential equations (ODEs). A liquid state machine (Maass and Markram 2004) is an echo state network with spiking neurons. ODEs in complex topology are similar to the lattice differential equations, but the connectivity is random. Moreover, computation in dynamical systems may be carried out in physical substrates (Tanaka et al. 2019), such as in-vitro networks of biological neurons (Aaser et al. 2017) or in nanoscale materials (Broersma et al. 2017). Finding the correct abstraction for the computation in a dynamical system, e.g. CA, is still an open research problem (Nichele et al. 2017).

One of our goals is to simulate all of these computing systems in a single general framework. Since generalization affects performance, we counterbalance it by using an optimized parallel library, such as the TensorFlow deep neural network framework (Abadi et al. 2016). To be able to exploit this library, a dynamical system is represented by a weighted adjacency matrix, such as a graph, and calculated as an artificial neural network, then taking advantage of the library’s optimization. Moreover, the weighted adjacency matrix of a dynamical system with complex dynamics is normally sparse. Thus, the choice of TensorFlow is advantageous because of its optimized methods and data types for sparse matrices or tensors. Another goal is to tune dynamical systems to reach the critical point at the “edge of chaos”, criticality, or even to search for systems with self-organized criticality. Systems in self-organized criticality may be better suited for performing useful computation in reservoir computing. To accomplish our goals, the presented general framework for dynamical systems, called EvoDynamic^1^, aims at evolving (i.e., using evolutionary algorithms) the connectivity, update and learning rules of sparsely connected networks to improve their usage for reservoir computing guided by the echo state property, fading memory, state trajectory, and other quality measurements. Such improvement of reservoirs is similarly applied in (Subramoney et al. 2019), where the internal connectivity of a reservoir is trained to increase its performance to several tasks. To verify that, we evolved three different stochastic dynamical systems, namely a cellular automaton, random Boolean network, and echo state network, towards criticality using a genetic algorithm. In the previous works (Pontes-Filho et al. 2019a, b), only cellular automaton is investigated and the fitness function for the genetic algorithm in (Pontes-Filho et al. (2019a) is less effective than the one proposed in this work. The evolution of these three stochastic dynamical systems was guided by fitting a power-law model into the distributions of avalanche size and duration. Moreover, for future work, evolution will model the dynamics and behavior of physical reservoirs, such as in-vitro biological neural networks interfaced with microelectrode arrays, and nanomagnetic ensembles. These two substrates have real applicability as reservoirs. For example, the former substrate is applied to control a robot, in effect making it a cyborg, a closed-loop biological-artificial neuro-system (Aaser et al. 2017), and the latter possesses computation capability as shown by a square lattice of nanomagnets (Jensen et al. 2018). These substrates are the main interest of the SOCRATES project (https://www.ntnu.edu/socrates) which aims to explore a dynamic, robust, and energy efficient hardware for data analysis.

This paper is organized as follows. Section 2 describes our method of computing dynamical systems in a generalized manner and the approach of evolving three stochastic dynamical systems towards criticality. Section 3 presents the results obtained from the methods. Section 4 discusses the experimental results. Section 5 states the initial advances and future plan for the EvoDynamic framework and Sect. 6 concludes this paper.

## Methods

There are two main methods described in this section. One method is to simulate dynamical systems in a general manner, which is very similar to simulating an artificial neural network, and no training is needed. The other method is to evolve three stochastic dynamical systems towards criticality. The three systems are based on cellular automata, random Boolean networks, and echo state networks, respectively.

### General framework for dynamical systems

Generalization is necessary to be able to simulate several dynamical systems with a single implementation. Therefore, our idea is to procedurally modify the computation of an artificial neural network to fit the dynamics of the desired dynamical system. In order to do that, modifications are introduced in the weighted adjacency matrix ${\mathbf {A}}$ and the mapping function *f*. ${\mathbf {A}}$ and *f* are analogous, respectively, to the weight matrix and activation function of artificial neural networks. The weighted adjacency matrix ${\mathbf {A}}$ and the mapping function *f* are used to compute the next state in time $t+1$ from the current state in time *t* of the components of the dynamical system that are called cells ${\mathbf {c}}$. The equation for that is

1 $$\begin{aligned} {\mathbf {c}}_{t+1} = f({\mathbf {A}}\cdot {\mathbf {c}}_{t}). \end{aligned}$$

This is similar to the equation of the forward pass of an artificial neural network but without the bias. The next states of the cells ${\mathbf {c}}_{t+1}$ are calculated from the result of the mapping function *f* which receives as argument the dot product between the weighted adjacency matrix ${\mathbf {A}}$ and the current states of the cells ${\mathbf {c}}_t$. The vector ${\mathbf {c}}$ is always a column vector of size $len({\mathbf {c}})\times 1$, and ${\mathbf {A}}$ is a matrix of size $len({\mathbf {c}})\times len({\mathbf {c}})$. Hence the result of ${\mathbf {A}}\cdot {\mathbf {c}}$ is also a column vector of size $len({\mathbf {c}})\times 1$ as ${\mathbf {c}}$.

Dynamical systems that possess a critical regime are often sparsely connected networks. Since the EvoDynamic framework is implemented on TensorFlow, the data type of the weighted adjacency matrix ${\mathbf {A}}$ is preferably a SparseTensor. A dot product with such a data type can result in up to 9 times faster execution than the dense counterpart. However, this depends on the configuration of the tensors (or, in our case, the adjacency matrices) (https://www.tensorflow.org/api_docs/python/tf/sparse/sparse_dense_matmul).

The details of how this general framework is used for the three stochastic dynamical systems that are evolved towards criticality are described in the following sections.

#### Cellular automata in the general framework

The implementation of a cellular automaton in our general framework requires the procedural generation of the weighted adjacency matrix of its grid. In this way, any lattice type or multidimensional CAs can be implemented using our framework. Algorithm 1 generates the weighted adjacency matrix for one-dimensional CA, such as the elementary cellular automaton (Wolfram 2002), where *widthCA* is the width or number of cells of a unidimensional CA and the vector $\mathbf {neighborhood}$ describes the region around the center cell. The connection weights depend on the type of update rule as previously explained. For example, in the case of an elementary CA, $\mathbf {neighborhood}=[4\ 2\ 1]$ (acquired from (2)). *indexNeighborCenter* is the index of the center cell in the $\mathbf {neighborhood}$ whose starting index is zero. *isWrappedGrid* is a Boolean value that works as a flag for adding a wrapped grid or not. A wrapped grid for one-dimensional CA means that the initial and final cells are neighbors. With all these parameters, Algorithm 1 creates an adjacency matrix by looping over the indices of the cells (from zero to $numberOfCells-1$) with an inner loop for the indices of the neighbors. If the selected *currentNeighbor* is a non-zero value and its indices do not affect the boundary condition, then the value of *currentNeighbor* is assigned to the adjacency matrix ${\mathbf {A}}$ in the indices that correspond to the connection between the current cell in the outer loop and the actual index of *currentNeighbor*. Finally, this procedure returns the adjacency matrix ${\mathbf {A}}$.

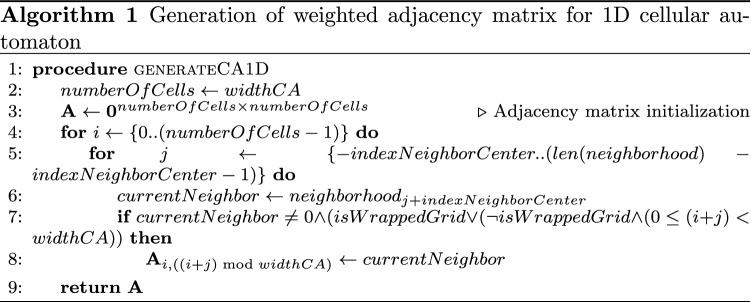



Minor adjustments need to be made to the algorithm to procedurally generate an adjacency matrix for 2D CA instead of 1D CA. Algorithm 2 shows the procedure for two-dimensional CA, such as Conway’s Game of Life. In this case, the height of the CA is an argument passed as *heightCA*. $\mathbf {Neighborhood}$ is a 2D matrix and $\mathbf {indexNeighborCenter}$ is a vector of two components meaning the indices of the center of $\mathbf {Neighborhood}$. This procedure is similar to the one in Algorithm 1, but it contains one more loop for the additional dimension.

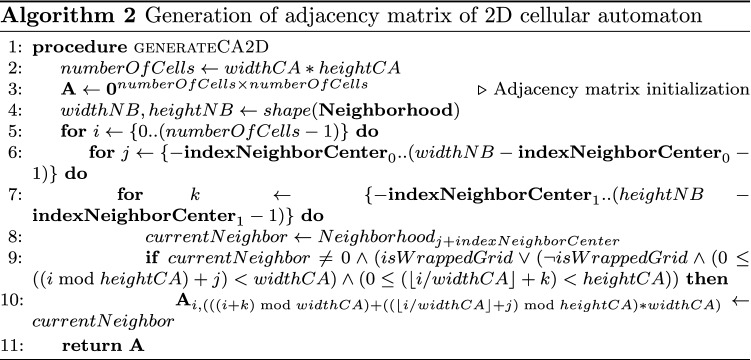



**Fig. 1:**
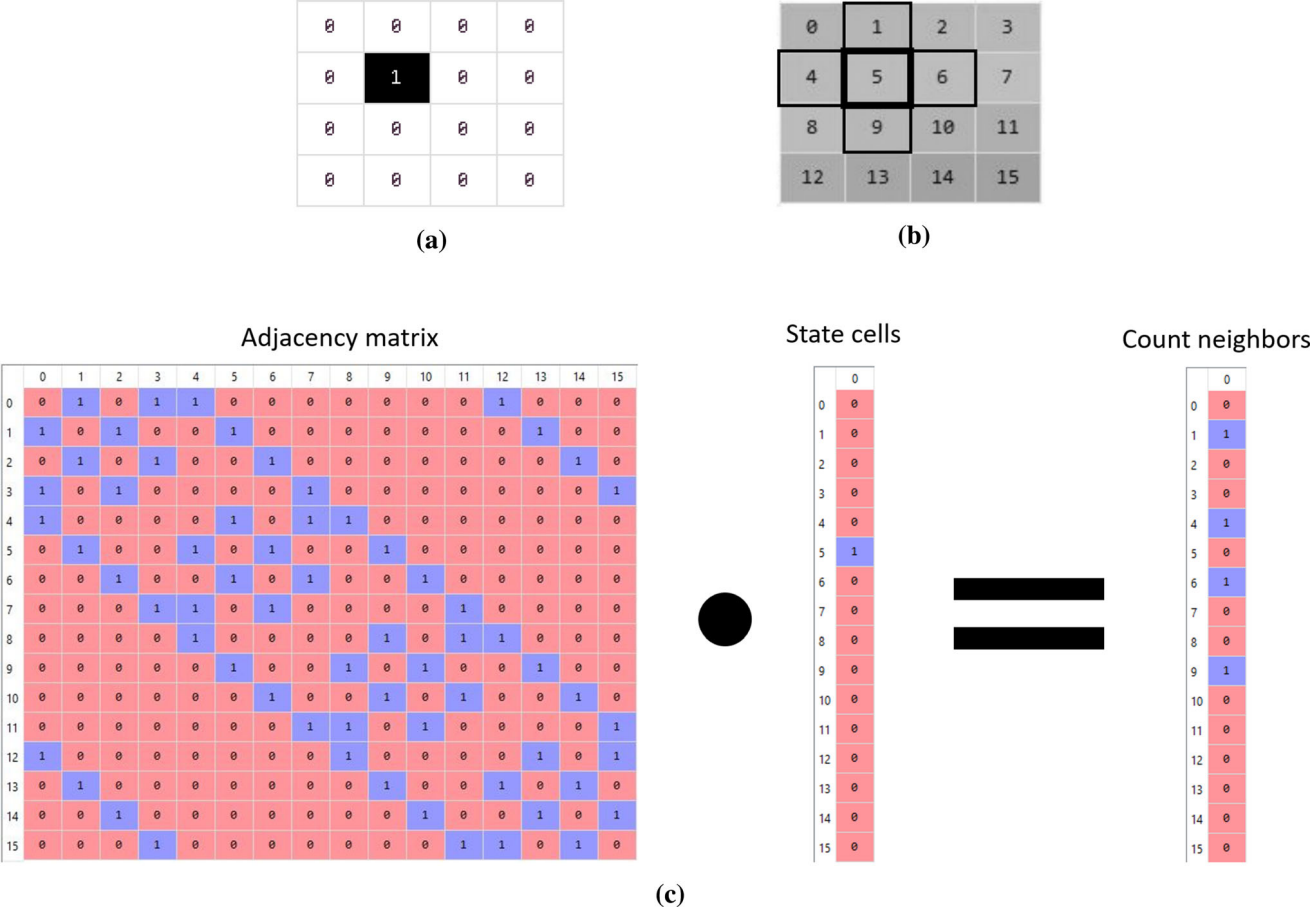
Example of using matrix multiplication for computing a 2D cellular automaton with 16 cells ($4\times 4$) and wrapped grid. **a** Example of the grid of cells with states. State 0 means dead or non-occupied cell and state 1 stands for alive or occupied cell. **b** Indices of the cells and von Neumann counting neighborhood of 2D CA where thick border means the current cell and thin border means the neighbors. **c** Illustration of matrix multiplication between adjacency matrix of the 2D CA and the state vector of the flattened 2D CA, resulting in a vector that contains the number of alive neighbors for each cell. Please note that an alive cell does not count itself as an alive neighbor

The update rule of the CA alters the weights of the connections in the adjacency matrix. For example, Conway’s Game of Life (Rendell 2002) is a CA whose cells have two states meaning “dead” (zero) or “alive” (one), and the update rule is based on the number of “alive” cells in the neighborhood. Therefore, for counting the number of alive “neighbors”, the weights in the adjacency matrix are one for connection and zero for no connection, as in an ordinary adjacency matrix. Such a matrix facilitates the description of the update rule for counting the number of “alive” neighbors because the result of the dot product between the adjacency matrix and the cell state vector is the vector that contains the number of “alive” neighbors for each cell. This is shown in Fig. 1 for a 2D CA of 16 cells ($4\times 4$), wrapped grids and modification in the original neighborhood (Fig. 1a), cells’ indices and von Neumann neighborhood (Fig. 1b), and its weighted adjacency matrix (acquired from Algorithm 2) which is used to compute the number of “alive” neighbors for this CA (Fig. 1c).

**Fig. 2:**
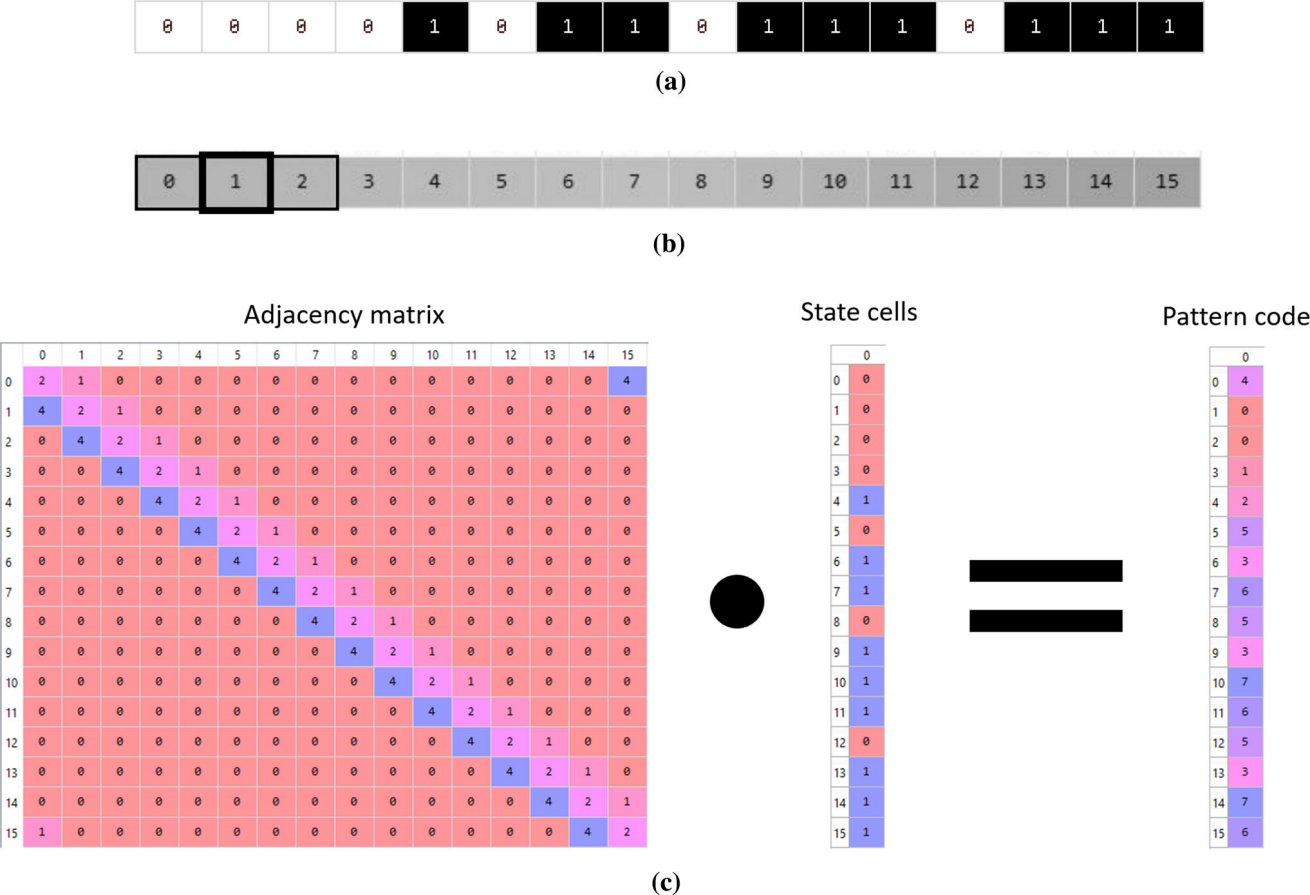
Example of using matrix multiplication for computing a 1D elementary cellular automaton with 16 cells and wrapped grid. **a** Example of the grid of cells with states. State 0 means dead or non-occupied cell and state 1 stands for alive or occupied cell. **b** Indices of the cells and 3-neighbors pattern neighborhood of 1D CA where thick border means the current cell and thin border means the neighbors. **c** Illustration of matrix multiplication between adjacency matrix of the 1D CA and the state vector of the 1D CA, resulting in a vector that contains the pattern code of the neighborhood for each cell. Important to consider that an alive cell counts itself as an alive neighbor and that is why the diagonal of the adjacency matrix is fulfilled with weight 2

Another example where the CA’s update rule affects the weighted adjacency matrix is when the pattern of the neighborhood influences the update rule, such as in an elementary cellular automaton (Wolfram 2002). To do that, each cell has its neighbors encoded as a *n*-ary string where *n* means the number of states that a cell can have. Hence, the weights of the connections with the neighbors are *n*-base identifiers and are calculated by

2 $$\begin{aligned} neighbor_{i}=n^{i},\forall {i}\in \{0..len(\mathbf {neighbors})-1\} \end{aligned}$$

where $\mathbf {neighbors}$ is a vector of the cell’s neighbors. In the adjacency matrix, each neighbor receives a weight according to (2). The result of the dot product with such a weighted adjacency matrix is a vector that consists of unique integers per neighborhood pattern. Thus, the mapping function is a lookup table from integer (i.e., pattern code) to next state. This is depicted in Fig. 2 for a 1D elementary cellular automaton of 16 cells and wrapped grids (Fig. 2a), cells’ indices and neighborhood (Fig. 2b), and its weighted adjacency matrix (acquired from Algorithm 1) being used to calculate the values for the mapping function (Fig. 2c).

The mapping function for CA is different from the activation function used for ANN. For CA, it contains the update rules that verify the vector returned by the dot product between the weighted adjacency matrix and the vector of states. Normally, the update rules of the CA are implemented as a lookup table from neighborhood to next state. In our implementation, the lookup table maps the resulting vector of the dot product to the next state of the central cell.

#### Random Boolean networks in the general framework

**Fig. 3:**
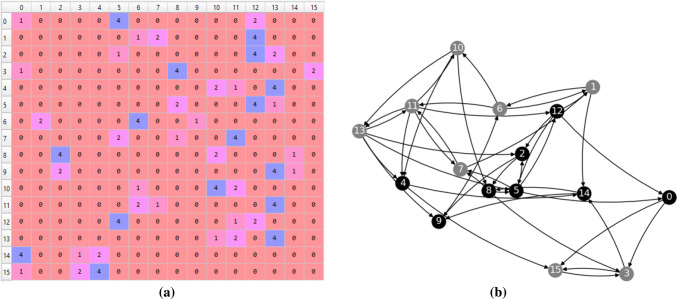
Example of a weighted adjacency matrix and graph for a random Boolean network with 16 cells and neighborhood of 3 cells. Self-connections are not shown in the graph

A random Boolean network (RBN) is an extension of cellular automata (Gershenson 2004) where the regular grid is replaced by random connections between the nodes or cells. An RBN has a similar update function to a CA whose cells consider the states of each of its neighbors, such as the neighborhood pattern of an elementary CA. Basically, a weighted adjacency matrix of a random Boolean network is acquired by shuffling the rows of the matrix for an elementary CA. Figure 3 illustrates the weighted adjacency matrix and the graph of a random Boolean network whose cells are randomly connected to three other cells. The difference between Figs. 2c and 3a shows how the method for elementary CA is adjusted for a random Boolean network.

#### Echo state networks in the general framework

**Fig. 4:**
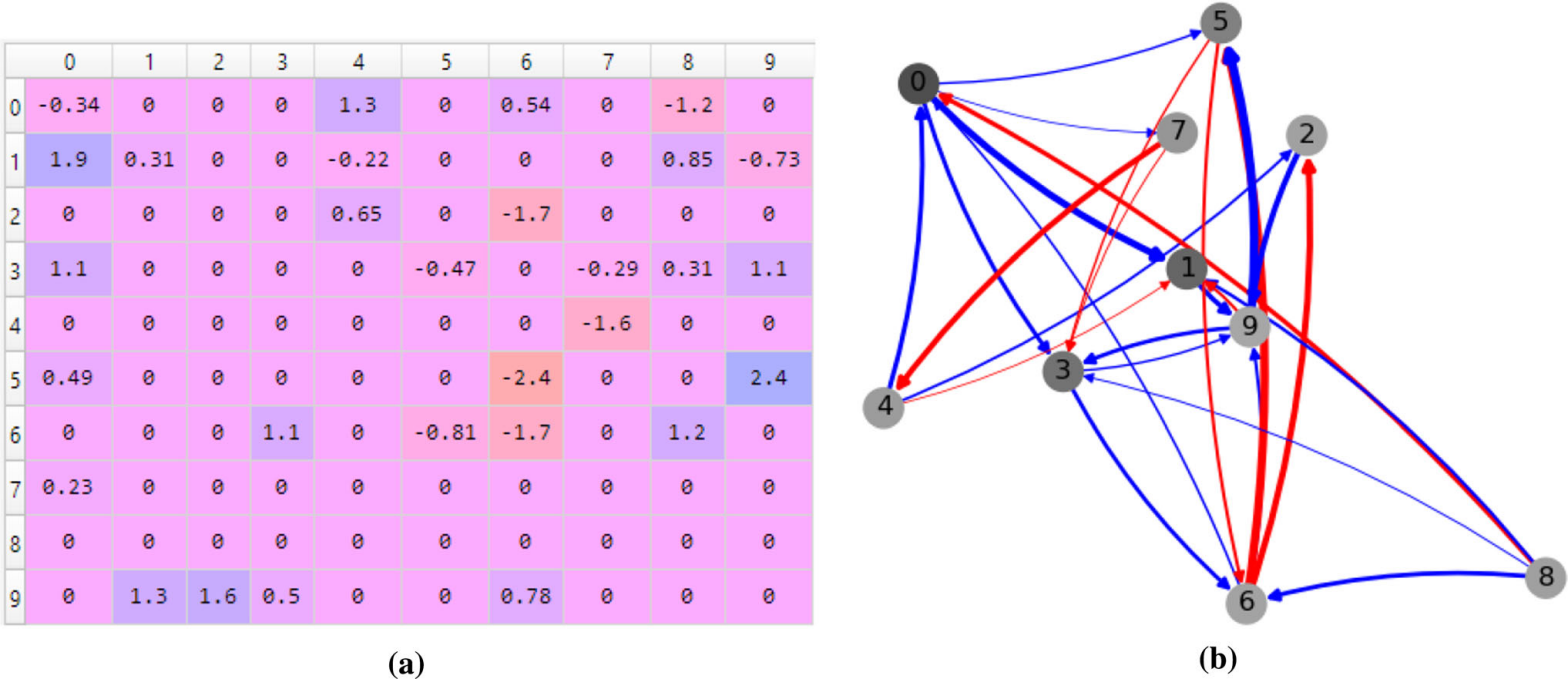
Example of a weighted adjacency matrix and graph for an echo state network with 10 cells or neurons. Red edges mean negative connections and blue edges mean positive connections. The thickness of the edges indicates the weight values. Self-connections are not shown in the graph

Our general framework for dynamical systems is based on the computation of artificial neural networks. Since an echo state network (ESN) (Jaeger and Haas 2004) is a type of artificial neural network, the weighted adjacency matrix is the usual weight matrix and the mapping function is one of the several activation functions that can be used for the neurons in an artificial neural network, such as sigmoid, hyperbolic tangent and rectified linear unit (LeCun et al. 2015). Note that in an ESN, the connection weights are randomly initialized. This is depicted in Fig. 4 where an echo state network of 10 cells or neurons are randomly connected with a certain sparsity. The color of the cells shows their states between 0 and 1 in grayscale. The edges are colored as red and blue to represent the negative and positive weights, respectively. The thickness of the edges is proportional to the weight value of the connections.

### Evolution of stochastic dynamical systems towards criticality

Using the previously explained general framework, we simulate three stochastic dynamical systems, namely cellular automata, random Boolean networks, and echo state networks. The evolution through genetic algorithm aims to find systems with criticality (Bak et al. 1987), in order to improve computational capacity (Langton 1990).

#### The stochastic dynamical systems

The first stochastic dynamical system towards criticality is a modified version of stochastic elementary cellular automata (SECA) introduced by Baetens et al. (2016). Our stochastic elementary cellular automaton is a modification of a 1D three neighbors elementary CA. Such modification is in the mapping function of the CA and the next state in time $t+1$ of the central cell $c_{i}$ is defined by a probability *p* to be 1 and a probability $1-p$ to be 0 for each of the eight different neighborhood patterns this CA has. Formally, probability *p* is represented by

3 $$\begin{aligned} p=P(c_{i,t+1}=1|N(c_{i,t})), \end{aligned}$$

where the neighborhood pattern $N(c_{i,t})$ is denoted as

4 $$\begin{aligned} N(c_{i,t})=(c_{i-1,t},c_{i,t},c_{i+1,t}). \end{aligned}$$

The second stochastic dynamical system that we evolve is based on random Boolean networks (RBNs). Basically, this is a modification of our stochastic cellular automata, but with the connectivity between the cells being random.

Our third and last stochastic dynamical system is based on echo state networks (ESNs). As its activation function, we use the sigmoid function denoted as

5 $$\begin{aligned} \text {sigmoid}(x)=\frac{1}{1+e^{-x}}. \end{aligned}$$

Since our echo state network is stochastic, the probability $p_{ESN}$ of next state being 1 is calculated by the sigmoid function in (5). This is given formally by

6 $$\begin{aligned} p_{ESN}=P(c_{i,t+1}=1)=\text {sigmoid}({\mathbf {A}}\cdot {\mathbf {c}}_{t}). \end{aligned}$$

#### Evolution through genetic algorithm

The evolution towards criticality is performed by a genetic algorithm. As described in the previous section, three different stochastic dynamical systems are evolved: CA, RBN and ESN. The genotype (or genetic code) for CA and RBN is the same. It contains one probability (value between 0.0 and 1.0) for each of the eight possible neighborhood configurations (three binary neighbors). The genome of the ESN consists of six values denoting mean and standard deviation of the weights of the positive connections ($mean_+$ and $std_+$), mean and standard deviation of the negative connections ($mean_-$ and $std_-$), probability of positive connections ($prob+$), and *sparsity*. The range of $mean_+$ and $mean_-$ is between 0.2 and 4.0, the values of $std_+$ and $std_-$ are determined by $mean_+$ and $mean_-$, and their genes $geneStd_+$ and $geneStd_-$ (values between 0.0 and 1.0). The equations for $std_+$ and $std_-$ are

7 $$\begin{aligned}&\mathrm{std}_+=0.2 \times \mathrm{mean}_+*\mathrm{geneStd}_+, \end{aligned}$$

8 $$\begin{aligned}&\mathrm{std}_-=0.2 \times \mathrm{mean}_-*\mathrm{geneStd}_-. \end{aligned}$$

The standard deviation values have a minimum of 0.0 and a maximum of $20\%$ of their corresponding mean. Such a maximum value for the standard deviation reduces the chances of sampling negative weights from the positive weight normal distribution, and vice-versa. However, in case this occurs, the absolute function is applied.

The fitness function which guides the stochastic dynamical systems towards criticality mainly verifies whether the probability distributions of avalanche size and duration follow a power-law distribution. The avalanche size and duration are acquired by the cluster size of identical states, which means the number of repetitions of a state that happened consecutively without the interruption of another state. The avalanche size stands for the clusters in the states in the same time-step and the avalanche duration consists of the clusters in the same cell through the time-steps of the simulation. The power-law distribution verification of the probability distributions of avalanche size and duration can be done in several ways. In our task, evolution is based on the verification of linearity in a log-log plot and the model comparison between power-law and exponential by the log-likelihood ratio (Clauset et al. 2009). The model comparison is an addition to the previous version of the fitness function for criticality in (Pontes-Filho et al. 2019a), which facilitates the convergence towards such a goal. After the evolution is completed, we test the best genome or individual with goodness-of-fit tests based on the Kolmogorov-Smirnov (KS) statistic (Clauset et al. 2009). To do that, the *p*-value of goodness-of-fit test is calculated using 1000 randomly generated data with 10,000 samples applying the power-law model estimated by maximum likelihood estimation method with minimum *x* of the distribution fixed to 1. The *p*-value measures the percentage of the KS statistic of the generated data when it is greater (worse) than the KS statistic of the empirical distribution. Therefore, a *p*-value of 1.0 or 100% is the best possible value and, to be accepted as power-law, the *p*-value must be greater than 0.1 (Clauset et al. 2009). The fitness function does not have goodness-of-fit test because it is computationally intensive. In our code, the log-likelihood ratio, generation of data from power-law model, and maximum likelihood estimation method are imported from the powerlaw Python library (Alstott et al. 2014).

The fitness function, used during evolution to calculate the genome’s fitness score, estimates the power-law model of the four distributions (avalanche size and duration for the state 0 and 1) acquired from the simulation of the stochastic binary dynamical system produced by the genome. The simulation runs 1,000 time-steps of a system with 1000 cells. The power-law model estimation is performed by linear fitting of the first 10 points of the log-log plot using least squares regression, which was verified to be unbiased and gives a fast and acceptable estimation of the slope of the power-law distribution (Goldstein et al. 2004). Their power-law models and empirical probability distributions are subsequently compared with the KS statistic and coefficient of determination (Wright 1921). The advantage of using the KS statistic with a model estimated by a linear 10-points fitting is that it reports a large error when the empirical distribution does not follow a power-law distribution. Another objective in the fitness function is the number of non-zero bins of size one in the raw histogram (empirical probability distribution). The number of non-zero bins is then normalized by dividing it with the maximum number of bins, which is 1000 for our case because 1000 cells are simulated through 1000 time-steps. Another objective is the percentage of unique states during the simulation (value between 0.0 and 1.0). In summary, the fitness function has scores calculated from the four probability distributions, which are the normalized number of non-zero bins *bin*; coefficient of determination $R^2$ of complete linear fitting; and KS statistic *D*. All these values are vectors of four elements. The fitness score *s* for those objectives is then calculated by the following equations:

9 $$\begin{aligned} bin_{s}&= {} \text {tanh}(5*(0.9*\text {max}(bin)+0.1*\text {mean}(bin))), \end{aligned}$$

10 $$\begin{aligned} R^2_{s}& {} = \text {mean}(R^2), \end{aligned}$$

11 $$\begin{aligned} D_{s} & {} = \text {exp}(-(0.9*\text {min}(D)+0.1*\text {mean}(D))). \end{aligned}$$

The fitness score which is based on the simulation result is the percentage of unique states, which is denoted by

12 $$\begin{aligned} unique_{s}=\frac{\#uniqueStates}{\#timesteps}. \end{aligned}$$

The Eqs. (9)–(12) are all objective values for calculating the temporary fitness score $s_{temp}$. Those values are real numbers between zero and one. Some important scores are squared, such as $R^2_{s}$ and $D_{s}$. The following equation denotes how the temporary fitness score $s_{temp}$ is calculated:

13 $$\begin{aligned} s_{temp}=bin_{s}+(R^2_{s})^2+(D_{s})^2+unique_{s}. \end{aligned}$$

The final fitness score includes the log-likelihood ratio which compares the power-law model with the exponential model for estimating the probability distribution. This process is computationally intensive. Therefore, such a score is only computed when the temporary fitness score $s_{temp}$ reaches a certain value. If the $s_{temp}$ is greater than this threshold value of 3.5, then the log-likelihood ratio is calculated for the four distributions and stored in the vector *l*. The log-likelihood ratio which is not trustworthy (*p*-value of ratio greater or equal to 0.1) are ignored (set as zero). The score for the log-likelihood ratio $l_s$ is then calculated by

14 $$\begin{aligned} l_{s}=\text {sigmoid}(10^{-2}*(0.9*\text {max}(l)+0.1*\text {mean}(l))). \end{aligned}$$

After describing all the objectives and their scores of our fitness function, the final equation is

15 $$\begin{aligned} s = {\left\{ \begin{array}{ll} s_{temp}+l_{s}, &{}s_{temp}>3.5\\ s_{temp}, &{}\text {otherwise.} \end{array}\right. } \end{aligned}$$

The configuration of the genetic algorithm consists of 40 individuals evolving through 100 generations. We run the genetic algorithm five times for each of the three dynamical systems. The goal of the genetic algorithm is to maximize the fitness score. The selection of two parents is done by deterministic tournament selection of two individuals (Goldberg and Deb 1991), which means that all individuals are assigned for the tournaments. Afterwards, the crossover between the genomes of the selected parents may occur with probability 0.8, and then each gene can be exchanged with probability 0.5. After that, a mutation can modify a gene with probability 0.1. This mutation adds a random value from a normal distribution with mean and standard deviation equal to 0 and 0.2, respectively. The mating process of the two parents produces an offspring of two new individuals who replace the parents in the next generation.

## Experimental results

The results of the methods described for a general framework for dynamical systems are described and explained in this section. The results of the genetic algorithm for criticality in three stochastic dynamical systems are also described and explained.

### Results of general framework

Figure 1 shows the result of Algorithm 2. It describes a wrapped 2D CA (similar to Game of Life but with a lower number of neighbors) and shows the resulting adjacency matrix. Figure 1a illustrates the desired two-dimensional CA with 16 cells (i.e., $widthCA=4$ and $heightCA=4$). Figure 1b presents the von Neumann neighborhood without considering the center cell (Toffoli and Margolus 1987) which is used for counting the number of “alive” neighbors (the connection weights are only zero and one, and defined by $\mathbf {Neighborhood}$ argument of Algorithm 2). It also shows the index distribution of the CA whose order is preserved after flattening it to a column vector. Figure 1c contains the generated adjacency matrix of Algorithm 2 for the described 2D CA. Figure 1b shows an example of a central cell with its neighbors, the index of this central cell is 5 and the row index 5 in the adjacency matrix of Fig. 1c presents the same neighbor indices, i.e., 1, 4, 6 and 9. Since this is a symmetric matrix, the columns have the same connectivity of the rows. This implies that the neighborhood of a cell considers the cell itself as a neighbor. Therefore, the connections are bidirectional and the adjacency matrix represents an undirected graph. The wrapping effect is also observable. For example, the neighbors of the cell index 0 are 1, 3, 4 and 12. So the neighbors 3 and 12 are the ones that the wrapped grid allowed to exist for cell index 0.

Figure 2 contains the result of Algorithm 1 together with (2). It illustrates a wrapped elementary CA and its generated weighted adjacency matrix. Figure 2a shows the appearance of the desired elementary CA with 16 cells ($widthCA=16$). Figure 2b describes its 3-neighborhood pattern and the indices of the cells. Figure 2c shows the result of Algorithm 1 with the neighborhood calculated by (2) for pattern matching in the activation function. In Fig. 2c, we can verify that the left neighbor has weight equal to 4 (or $2^2$ for the most significant bit), central cell weight is 2 (or $2^1$) and the right neighbor weight is 1 (or $2^0$ for the least significant bit) as defined by (2). Since the CA is wrapped, we can notice in row index 0 of the adjacency matrix in Fig. 2c that the left neighbor of cell 0 is cell 15, and in row index 15 that the right neighbor of cell 15 is cell 0.

Figure 3 sets out the result of (2). The neighborhood is defined as *n*-ary string for the purpose of identifying the states of each neighbor. The neighbors of a cell are selected randomly and are represented in the matrix row of the cell’s index. Therefore, the neighbor identifiers, which are in this case 1, 2 and 4, are assigned to their corresponding neighbor.

### Results of evolving dynamical systems towards criticality

**Fig. 5:**
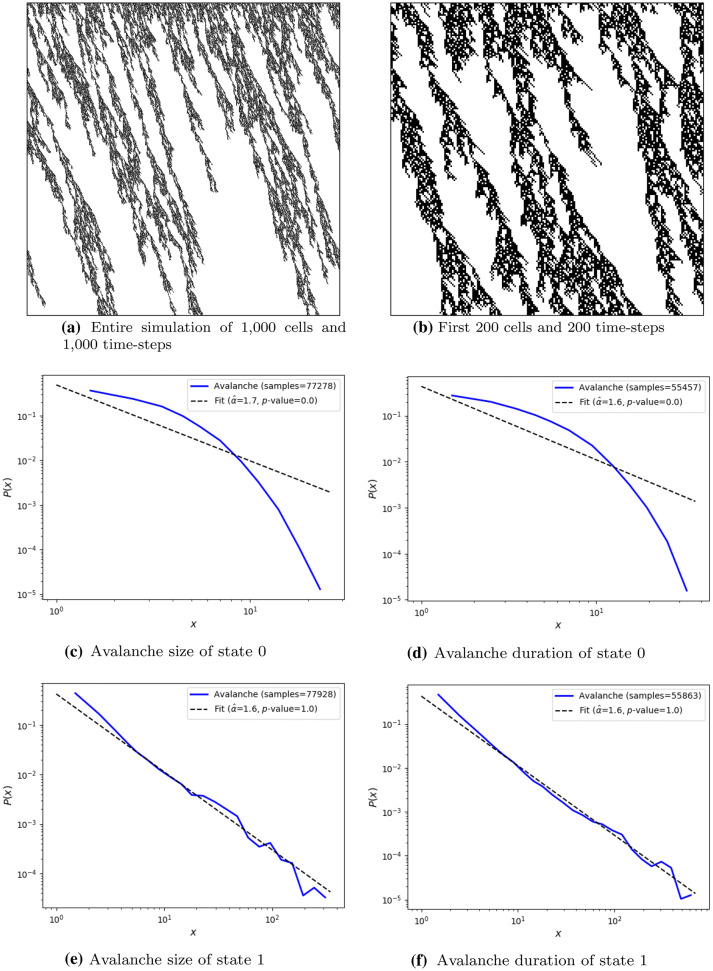
Test sample of the 2nd best evolved stochastic elementary CA of 1,000 cells (horizontal axis) randomly initialized with wrapped boundaries and run through 1,000 time-steps (vertical axis), and its avalanche size and duration of the two states 0 (black) and 1 (white). Fitness score of this simulation is 4.383

After five independent runs of the CA evolution, the best genome solutions turn out to be unstable, i.e., the test score of the best genome differs significantly when compared to the score obtained during evolution. For this reason, the 2nd best solution is selected, as its test score shows stable results. The genome of the stable solution is presented in Table 2. Its fitness score and all objective scores during evolution and testing are in Table 3. It can be observed that the CA results are stable because of the low standard deviation of the scores in the five testing executions. This is further supported by the mean test score being larger than the score during evolution. Fig. 5 contains the image produced by the entire simulation, by the first 200 cells and 200 time-steps, and by the four probability distributions with their corresponding power-law model estimated by maximum log-likelihood and *p*-value of the goodness-of-fit test. The empirical probability distributions (depicted in Figs. 5c–f) which fit to a power-law model are the probability distributions of avalanche size and duration of state 1 (Figs. 5e and 5f). This can be concluded quantitatively by the *p*-values of their goodness-of-fit test being equal to 1.0, which to be considered a power-law distribution *p*-value must be greater than 0.1 (Clauset et al. 2009). Moreover, the large number of samples confirms that these *p*-values are reliable; and qualitatively by the similarity of their power-law estimated models (black dashed line) and the empirical distributions (blue solid line). Therefore, we can conclude that the presented CA shows criticality for state 1.

**Table 2:** Selected 2nd best CA in fitness score

Neighborhood $N(c_{i,t})$	Probability *p*
(0,0,0)	0.394221
(0,0,1)	0.094721
(0,1,0)	0.239492
(0,1,1)	0.408455
(1,0,0)	0.000000
(1,0,1)	0.730203
(1,1,0)	0.915034
(1,1,1)	1.000000

**Table 3:** Fitness score of the selected 2nd best CA. Testing simulations were performed 5 times and “std.” stands for standard deviation. Numbers are rounded to three decimal places

Objective	Evolution score	Test score mean	Test score std.
$R^2_{s}$	0.870	0.866	0.006
$D_{s}$	0.961	0.961	0.003
$bin_{s}$	0.966	0.980	0.007
$unique_{s}$	1.000	1.000	0.000
$l_{s}$	0.728	0.733	0.016
*s*	**4.376**	**4.387**	**0.015**

**Fig. 6:**
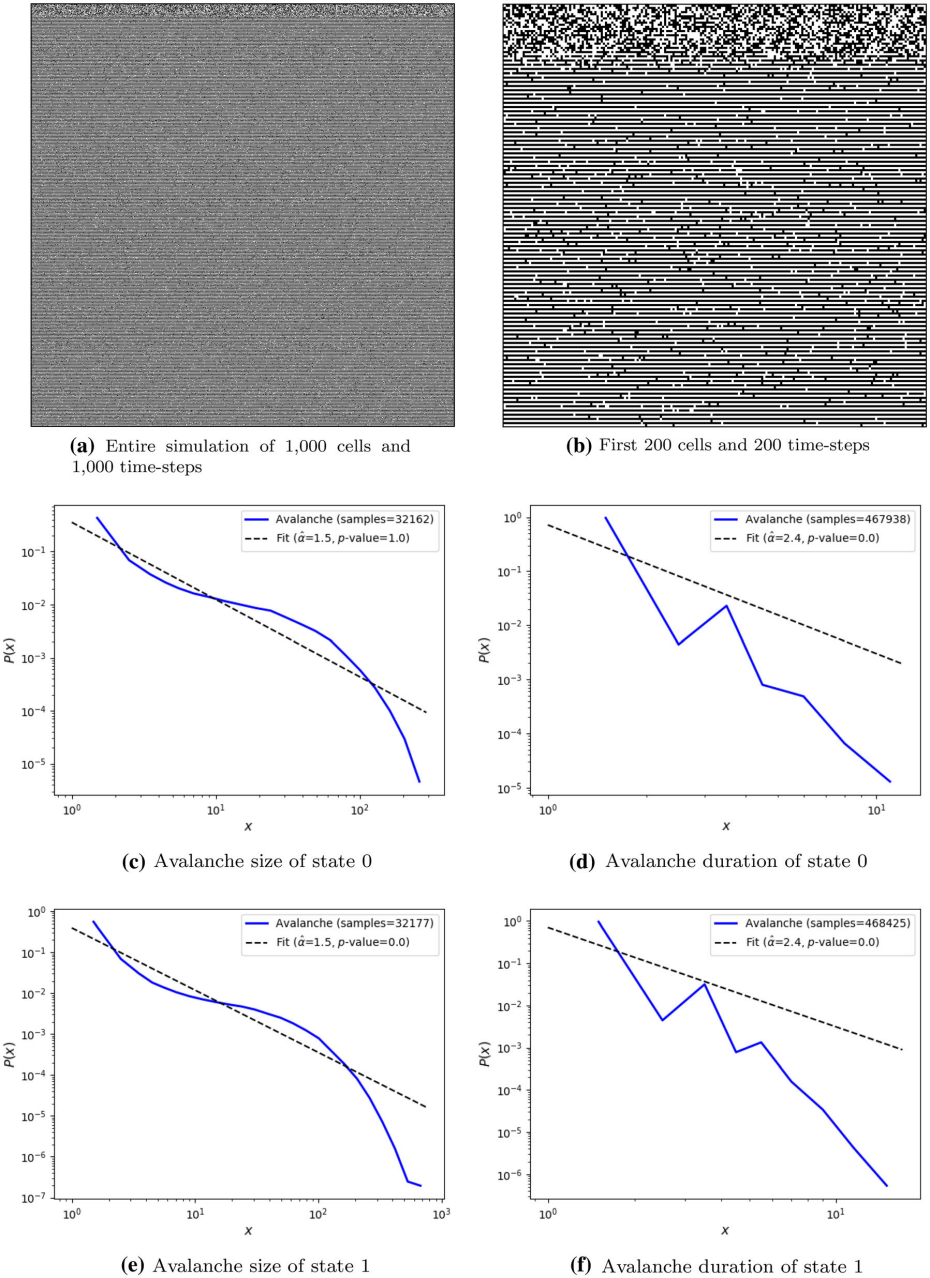
Test sample of the 1st best evolved stochastic RBN of 1000 cells (horizontal axis) randomly initialized and run through 1000 time-steps (vertical axis), and its avalanche size and duration of the two states 0 (black) and 1 (white). Fitness score of this simulation is 3.315

Repeating the same procedure used for CA, the RBN’s 1st best individual presented a high score as the 2nd best CA score, but the 1st best RBN is unstable. The following best individuals are also showing instability. Hence, we keep the selection of the 1st best individual. Table 4 contains the genome of the selected RBN. Table 5 has the scores acquired during evolution and the mean and standard deviation of the five test runs. Figure 6 illustrates the simulation of the RBN and their avalanche distributions. It can be noted that none of the distributions qualitatively resembles a power-law, but Fig. 6c shows the distribution of avalanche size of state 0 which has a *p*-value of goodness-of-fit test equal to 1.0 which means that it is classified as power-law according to this evaluation method. Nevertheless, if we consider that such RBN does not achieve criticality, we can hypothesize that the random connections may be a bottleneck to achieving this behavioral regime while, with a regular grid, CA more easily achieved a critical behavior through its evolution.

**Table 4:** Selected 1st best RBN in fitness score

Neighborhood $N(c_{i,t})$	Probability *p*
(0,0,0)	1.000000
(0,0,1)	0.844143
(0,1,0)	0.950141
(0,1,1)	0.314001
(1,0,0)	0.527704
(1,0,1)	0.314433
(1,1,0)	0.109056
(1,1,1)	0.015699

**Table 5:** Fitness score of the selected 1st best RBN. Testing simulations were performed 5 times and “std.” stands for standard deviation. Numbers are rounded to three decimal places

Objective	Evolution score	Test score mean	Test score std.
$R^2_{s}$	0.886	0.905	0.002
$D_{s}$	0.953	0.867	0.050
$bin_{s}$	0.867	0.864	0.007
$unique_{s}$	1.000	1.000	0.000
$l_{s}$	0.706	0.145	0.291
*s*	**4.266**	**3.583**	**0.353**

**Fig. 7:**
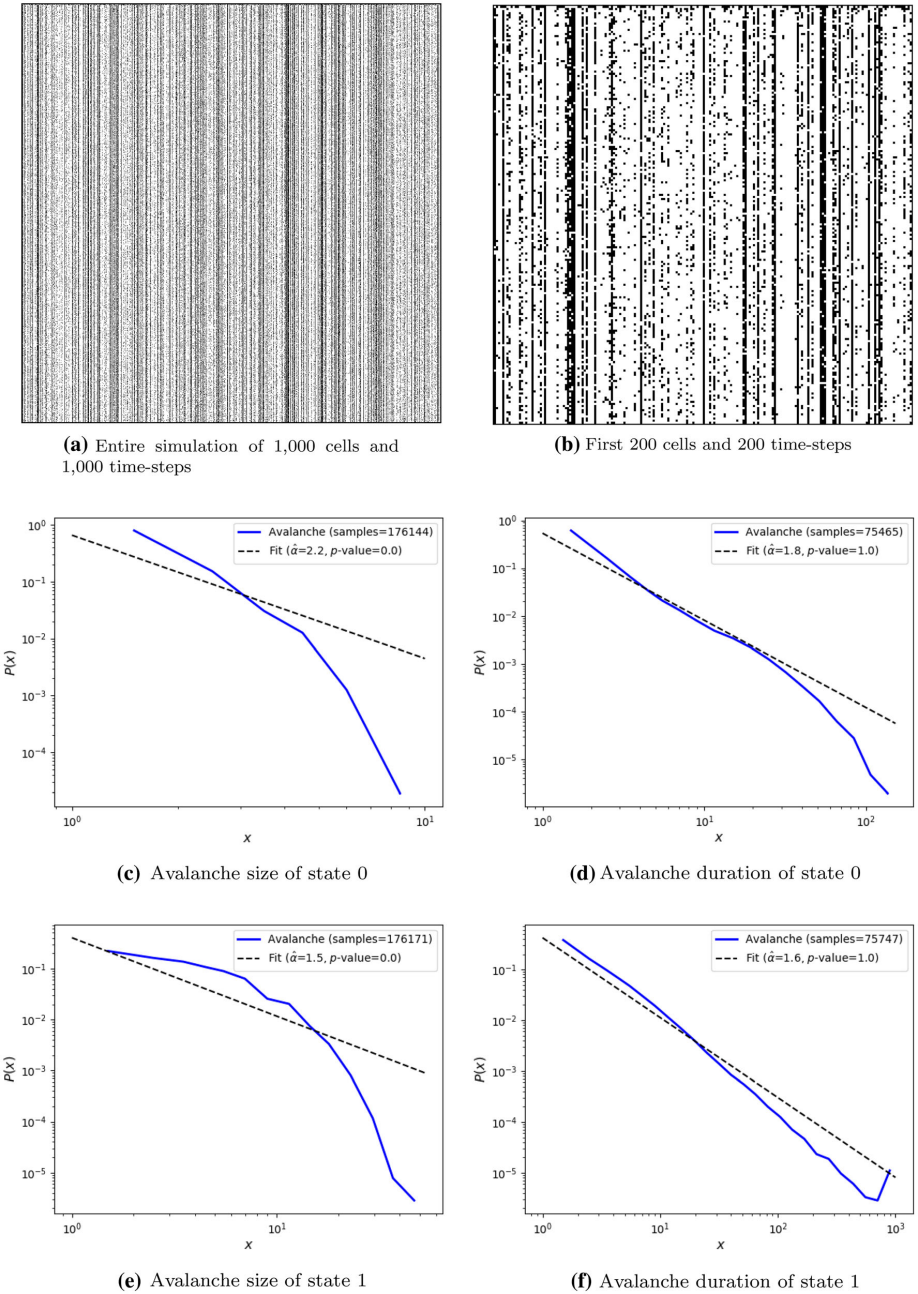
Test sample of the 2nd best evolved stochastic ESN of 1000 cells (horizontal axis) randomly initialized and run through 1,000 time-steps (vertical axis), and its avalanche size and duration of the two states 0 (black) and 1 (white). Fitness score of this simulation is 4.158

The ESN results are presented in Table 6, Table 7, and Fig. 7. The 1st best ESN was found to be unstable as the 1st best CA. Therefore, the selected genome is the 2nd best which presents stable results. The CA and ESN’s selected best individuals possess two distributions which are considered power-laws by the *p*-value of goodness-of-fit test. However, the ESN’s avalanche distributions with *p*-value equal to 1.0 are the avalanche duration of state 0 and 1. This means that avalanches that present criticality do not occur within the states through the simulation. The criticality occurs only by combining the cluster sizes of each of the cells in the system during the simulation.

**Table 6:** Selected 2nd best ESN in fitness score

Genome	Value
$mean_+$	4.000000
$std_+$	0.800000
$mean_-$	0.100000
$std_-$	0.007792
$prob+$	0.064934
*sparsity*	0.963955

**Table 7:** Fitness score of the selected 2nd best ESN. Testing simulations were performed 5 times and “std.” stands for standard deviation. Numbers are rounded to three decimal places

Objective	Evolution score	Test score mean	Test score std.
$R^2_{s}$	0.891	0.891	0.006
$D_{s}$	0.903	0.885	0.038
$bin_{s}$	0.968	0.965	0.004
$unique_{s}$	1.000	1.000	0.000
$l_{s}$	0.613	0.479	0.239
*s*	**4.190**	**4.024**	**0.282**

We consider that the evolved stochastic dynamical system achieved criticality when at least one of the probability distributions of the avalanche size and duration is a power-law distribution. That is, quantitatively evaluated by the *p*-value of the goodness-of-fit test. Table 8 contains the mean and standard deviation of the *p*-value of the four avalanche distributions. Through this result, we can affirm that two out of the four presented distributions for the CA and ESN show a power-law distribution, i.e., at criticality. The presented results also show that the tested RBN possesses only one avalanche distribution which can be considered as a power-law; the avalanche size distribution of state 0. Moreover, the *p*-value of this distribution of RBN is not as stable as the two critical avalanche distributions of CA and ESN with mean equaling 1.0 and standard deviation equaling 0.0.

**Table 8:** Goodness-of-fit test of the three evolved stochastic dynamical systems. Avalanche size (AS) and avalanche duration (AD) are followed by the state from which they were calculated. Testing simulations were performed 5 times and *p*-values are denoted as “mean ± standard deviation”. The *p*-values in bold are the ones that are considered a power-law distribution. So, $p\text {-value}>0.1$

System	*p*-value of AS	*p*-value of AD	*p*-value of AS	*p*-value of AD
	of state 0	of state 0	of state 1	of state 1
CA	0.0 ± 0.0	0.0 ± 0.0	**1.0 ± 0.0**	**1.0 ± 0.0**
RBN	**0.969 ± 0.021**	0.0 ± 0.0	0.0 ± 0.0	0.0 ± 0.0
ESN	0.0 ± 0.0	**1.0 ± 0.0**	0.0 ± 0.0	**1.0 ± 0.0**

## Discussion

The results of the evolution of the three stochastic dynamical systems show the potential of such systems to produce criticality. Evaluating these systems, we can deduce that the stochastic cellular automaton is the system that can become critical most easily. This is followed by the stochastic echo state network, which in our results presented an unexpected behavior where the only avalanche distributions that can be considered critical are the two avalanche duration distributions. This result is unexpected if compared to the presented CA, which presents only one state (state 1) as critical in both avalanche size and duration. The stochastic random Boolean network is very similar to the stochastic CA, with the difference that the connectivity is randomized instead of regular. Such modification may make it more difficult to evolve the RBN into a critical system behavior. The RBN only shows a single critical avalanche distribution and is not stable like the two critical avalanche distributions of CA and ESN.

## Ongoing and future applications with EvoDynamic

The generalization of representations for different dynamical systems presented in this work is beneficial for the further development of the EvoDynamic framework. Cellular automata, random Boolean networks, and echo state networks are already implemented in our Python library. The implementation of the other described dynamical systems in the EvoDynamic framework is ongoing. In addition, the EvoDynamic framework will incorporate the possibility to evolve the connectivity, the update rules and the learning rules of the dynamical systems, in order to allow the dynamical systems to be used efficiently for reservoir computing, as well as for physical substrate modeling. The introduced general representation facilitates the evolution of such systems and models through methods that measure the quality of a reservoir system or the similarity to a given input dataset. The following subsection will further document an additional method under development, which can be used to assess the quality of a dynamical system model or substrate for reservoir computing.

### State trajectory

**Fig. 8:**
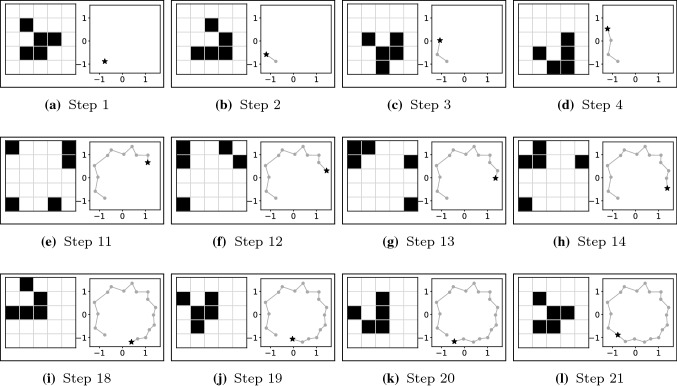
States of Conway’s Game of Life in a 5 x 5 wrapped lattice alongside their PCA-transformed state transition diagrams of the two first principal components. **a** Initial state is a glider. **a**–**d** Four first steps in this CA. **e**–**h** Four intermediate steps in this CA while reaching the wrapped border. **i**–**l** Four last steps in this CA before repeating the initial state and closing a cycle

A method that can guide dynamical systems’ evolutionary search is the state trajectory. This method can be used to cluster similar states for model abstraction and to measure the quality of the reservoir. For this purpose, a graph can be generated and analyzed by searching for attractors and cycles in the obtained state space. For visualization of the state trajectory, we apply principal component analysis (PCA) to reduce the dimensionality of the states considering the entire dynamical system simulation (each time-step produces a sample for PCA). An example of the produced visualization is depicted in Fig. 8, where every produced state is shown as a state transition diagram. The chosen dynamical system shown in the Figure is a CA using Conway’s Game of Life’s rules with 5 x 5 cells and wrapped boundaries. The CA is initialized with a glider configuration as the initial state (Fig. 8a) and, subsequently, the CA cycles over 20 unique states, as illustrated in the state transition diagram in Fig. 8l.

## Conclusion

In this work, a general framework for simulating dynamical systems is described, which utilizes the computation of artificial neural networks as a general method for executing different dynamical systems. The presented framework, called EvoDynamic, is built on the Tensorflow deep learning library, which allows better performance and parallelization while keeping a common general representation based on operations on sparse tensors. The application of this framework is used in the work herein to evolve three different dynamical systems, i.e., cellular automata, random Boolean networks, and echo state networks, towards criticality. The presented results are promising for CA and ESN evolution, while further analysis and experiments are required to confirm critical behavior in the evolved RBNs. As future work, our goal is to evolve dynamical systems towards self-organized criticality, i.e., a dynamical system that self-organizes into a critical state without the need to tune control parameters. Ongoing and future implementations of EvoDynamic are presented and discussed, such as the visualization and usage of state trajectories, as well as the possibility of physical substrate modeling. EvoDynamic is an open-source framework currently under development that primarily targets applications in reservoir computing and artificial intelligence. We envision that the generalization and parallelization of the described dynamical systems will enable our Python library to be widely used by the research community.

## Footnotes

1 EvoDynamic open-source repository on https://github.com/SocratesNFR/EvoDynamic.
